# Supplementary material for: Mapping hippocampal-cerebellar functional connectivity across the human adult lifespan
Source: Commun Biol. 2025 Nov 20;8:1619. doi: 10.1038/s42003-025-08972-2 (PMC12635369; doi:10.1038/s42003-025-08972-2)
Supplement: Supplementary file 2 — Supplementary Information [file 42003_2025_8972_MOESM2_ESM.pdf]

Supplementary information

Mapping hippocampal-cerebellar functional connectivity across the human adult lifespan

Kavishini Apasamy[1], Samuel C Berry[1,2], Marie-Lucie Read[2], Narender Ramnani[1]*, Carl J. Hodgetts[1,2] *

1. Department of Psychology, Royal Holloway, University of London, Egham, Surrey, TW20 0EX, UK
2. Cardiff University Brain Research Imaging Centre (CUBRIC), School of Psychology, Cardiff University, Cardiff CF24 4HQ, United Kingdom

* These authors jointly supervised this work

For correspondence: carl.hodgetts@rhul.ac.uk

Contents

- Supplementary Results
- Supplementary Figure 1

Supplementary Results

We conducted a supplementary second-level analysis in CONN to include Body Mass Index (BMI) and sex as covariates-of-no-interest in the ageing analysis (contrast vector: 0 -1 0 0), using a subset of participants with available BMI data (N = 425; see Methods, main text). As shown in Supplementary Figure 1, controlling for BMI and sex resulted in modest changes to the strength and spatial distribution of age-related hippocampal functional correlations within the cerebellum.

For the left hippocampal seed, age-related reductions were still observed along the border of cerebellar lobules HV and HVI, though fewer voxels extended into lobule HVIIA (Crus I; see Figure S1). The medial cluster – spanning Crus II, Lobule VIIIA and HVIIIB – was slightly more extensive when controlling for these additional covariates.

For the right hippocampal seed, the pattern of connectivity alterations continued to involve bilateral areas of lobule HVI, as well as Lobule HV in the contralateral hemisphere. Controlling for BMI and sex additionally revealed a new cluster at the border of lobules HVIIIA and VIIIB.

As in the primary analysis, seeding from the anterior and posterior hippocampus yielded broadly similar age-related connectivity patterns when controlling for additional covariates. For the anterior hippocampus, correlations were primarily localised to the primary fissure between lobules HV, with slight increases and decreases in cluster extent. The posterior hippocampus continued to show minimal age-related changes in functional connectivity, although clusters were slightly larger when controlling for BMI and sex in the model.

We also implemented separate second-level general linear models with BMI and sex as covariates-of-interest, controlling for age. For the negative contrast of BMI (contrast vector: 0 0 -1 0), we observed no significant BMI-related decreases in connectivity between left, right, anterior or posterior hippocampus and the cerebellum. For the positive BMI contrast (contrast vector: 0 0 1 0), only the anterior hippocampus showed a BMI-related increase in connectivity with the left cerebellar lobule VIIIA (coordinates: -12 -68 -50; 1 voxel; z = 4.98, t = 5.06). No other significant BMI-related increases in connectivity were observed between the left, right or posterior hippocampus and cerebellum when controlling for age and sex.

Finally, we also conducted positive and negative contrasts for sex, with females coded as 1 and males as 0. For the negative contrast (0 0 0 -1), which tested for reduced connectivity in females, we observed small clusters between the right hippocampus and right Crus II (coordinates: 6 -86 -42; 3 voxels; z = 5.26, t = 5.36), right hippocampus and left lobule IX (-8 -46 -40; 1 voxel; z = 5.23, t = 5.32), and between the anterior hippocampus and left lobule IX (-8 -46 -40; 4 voxels; z = 5.46, t = 5.56). No significant decreases in connectivity were found between the left or posterior hippocampus and the cerebellum for females. For the positive contrast (0 0 0 1), testing for increased connectivity in females, we found no significant voxels for any of the hippocampal seeds with the cerebellum..

Supplementary Figure 1

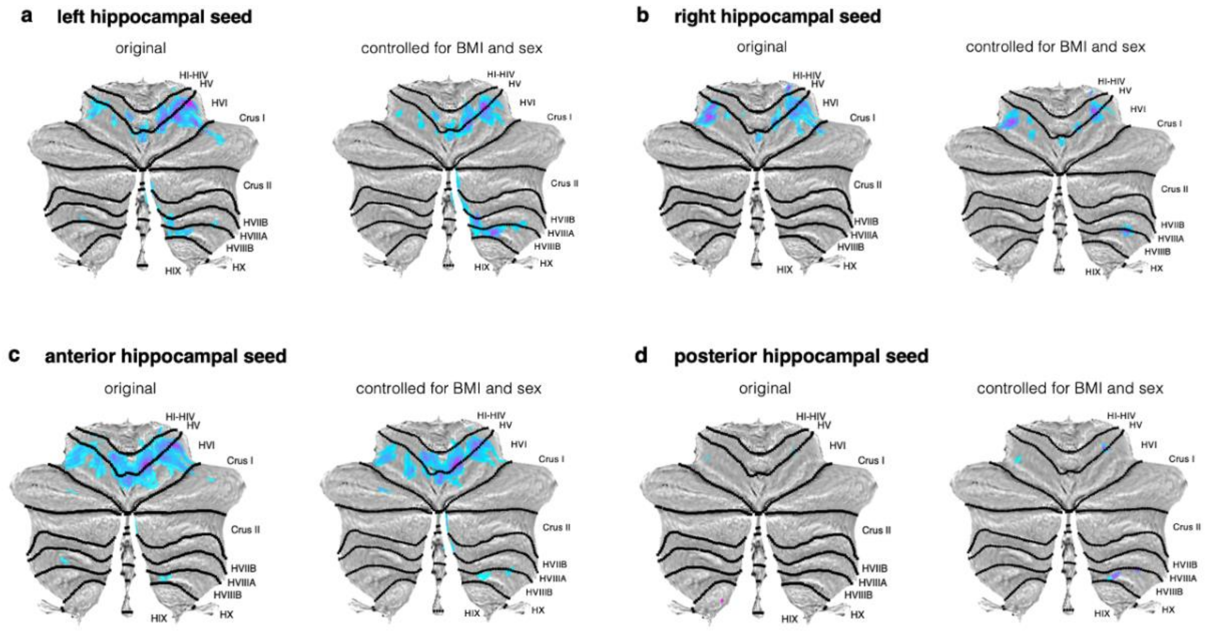

Supplementary Figure 1. Regions in the cerebellar cortex showing age-related decreases in functional connectivity with hippocampal seed regions and when controlling for BMI and sex. Thresholded SPM {T} maps are overlaid on cerebellar flatmaps and show the effect of ageing on cerebellar connectivity with (a) left, (b) right, (c) anterior and (d) posterior hippocampal seeds. The left of each panel shows the original flatmap (not controlled for BMI and sex; see main text) and the right shows the flatmap controlled for BMI and sex. No appreciable differences were observed when including these covariates.
