# Supplementary material for: Mapping hippocampal-cerebellar functional connectivity across the human adult lifespan
Source: Commun Biol. 2025 Nov 20;8:1619. doi: 10.1038/s42003-025-08972-2 (PMC12635369; doi:10.1038/s42003-025-08972-2)
Supplement: Supplementary file 3 — Reporting Summary [file 42003_2025_8972_MOESM3_ESM.pdf]

nature portfolio

Corresponding author(s): Carl J Hodgetts

Last updated by author(s): 26/09/2025

Reporting Summary

Nature Portfolio wishes to improve the reproducibility of the work that we publish. This form provides structure for consistency and transparency in reporting. For further information on Nature Portfolio policies, see our Editorial Policies and the Editorial Policy Checklist.

Statistics

For all statistical analyses, confirm that the following items are present in the figure legend, table legend, main text, or Methods section.

n/a	Confirmed	
☐	☒	The exact sample size (*n*) for each experimental group/condition, given as a discrete number and unit of measurement
☒	☐	A statement on whether measurements were taken from distinct samples or whether the same sample was measured repeatedly
☐	☒	The statistical test(s) used AND whether they are one- or two-sided<br>*Only common tests should be described solely by name; describe more complex techniques in the Methods section.*
☐	☒	A description of all covariates tested
☐	☒	A description of any assumptions or corrections, such as tests of normality and adjustment for multiple comparisons
☐	☒	A full description of the statistical parameters including central tendency (e.g. means) or other basic estimates (e.g. regression coefficient) AND variation (e.g. standard deviation) or associated estimates of uncertainty (e.g. confidence intervals)
☐	☒	For null hypothesis testing, the test statistic (e.g. F, t, r) with confidence intervals, effect sizes, degrees of freedom and P value noted<br>*Give P values as exact values whenever suitable.*
☒	☐	For Bayesian analysis, information on the choice of priors and Markov chain Monte Carlo settings
☒	☐	For hierarchical and complex designs, identification of the appropriate level for tests and full reporting of outcomes
☐	☒	Estimates of effect sizes (e.g. Cohen's *d*, Pearson's *r*), indicating how they were calculated

Our web collection on statistics for biologists contains articles on many of the points above.

Software and code

Policy information about availability of computer code

Data collection	The CamCAN dataset is publicly available at https://camcan-archive.mrc-cbu.cam.ac.uk/dataaccess/
Data analysis	All preprocessing, denoising, first- and second-level analyses were conducted using CONN (v. 22.a) default pipelines.

For manuscripts utilizing custom algorithms or software that are central to the research but not yet described in published literature, software must be made available to editors and reviewers. We strongly encourage code deposition in a community repository (e.g. GitHub). See the Nature Portfolio guidelines for submitting code & software for further information.

Data

Policy information about availability of data

All manuscripts must include a data availability statement. This statement should provide the following information, where applicable:

- Accession codes, unique identifiers, or web links for publicly available datasets
- A description of any restrictions on data availability
- For clinical datasets or third party data, please ensure that the statement adheres to our policy

We used structural and functional MRI data from the Cambridge Centre of Ageing and Neuroscience (CamCAN) study (Taylor et al., 2017).

All CamCAN MRI data was acquired using a 3T Siemens TIM Trio Scanner at the Medical Research Council (UK) Cognition and Brain Science Unit using a 32-channel

head coil. The data used in this study forms part of a larger scanning protocol (see https://camcan-archive.mrc-cbu.cam.ac.uk/dataaccess/ or Taylor et al., 2017 for more detail).

Research involving human participants, their data, or biological material

Policy information about studies with human participants or human data. See also policy information about sex, gender (identity/presentation), and sexual orientation and race, ethnicity and racism.

Reporting on sex and gender	Information about sex (biological attribute) was collected via self-report and considered in the study design (for further information about the data collection of CamCAN and informed consent procedures, see Taylor et al., 2017). Overall, the data analyses contained 242 males and 237 females. Sex was included as a covariate-of-interest and a covariate-of-no-interest, as requested by reviewers.
Reporting on race, ethnicity, or other socially relevant groupings	N/A
Population characteristics	Participants in the dataset analysed were between 18-88 years of age and cognitively healthy. Information about sex (biological attribute) was collected via self-report and considered in the study design (for further information about the data collection of CamCAN and informed consent procedures, see Taylor et al., 2017). Overall, the data analyses contained 242 males and 237 females. Sex was included as a covariate-of-interest and a covariate-of-no-interest, as request by reviewers.
Recruitment	For information about recruitment, please see Taylor et al., (2017).
Ethics oversight	Cambridgeshire Research Ethics Committee and Royal Holloway, University of London Ethics Committee

Note that full information on the approval of the study protocol must also be provided in the manuscript.

Field-specific reporting

Please select the one below that is the best fit for your research. If you are not sure, read the appropriate sections before making your selection.

☒ Life sciences ☐ Behavioural & social sciences ☐ Ecological, evolutionary & environmental sciences

For a reference copy of the document with all sections, see nature.com/documents/nr-reporting-summary-flat.pdf

Life sciences study design

All studies must disclose on these points even when the disclosure is negative.

Sample size	No statistical methods were used to predetermine the sample size. All available data were used excluding participants based on pre-selected exclusion criteria (see below). The final sample was made of 479 participants.<br><br>For supplementary analyses with BMI and sex, the final sample size was of 425 participants.
Data exclusions	Firstly, a visual inspection was conducted for about 25% of the cases, excluding any participants on poor data quality (visible ringing, motion, etc.). Following preprocessing and denoising, participants with volumes with framewise displacement above 0.5mm, or global BOLD signal above 3 SDs which were flagged as outliers were excluded. A further quality assurance, determined the final sample of 479 participants.<br><br>For the BMI and sex analyses, participants were excluded for data unavailability. The final sample made up of 425 participants.
Replication	N/A
Randomization	No groups were allocated and covariates of interest were not required to be controlled.
Blinding	N/A

Reporting for specific materials, systems and methods

We require information from authors about some types of materials, experimental systems and methods used in many studies. Here, indicate whether each material, system or method listed is relevant to your study. If you are not sure if a list item applies to your research, read the appropriate section before selecting a response.

Materials & experimental systems

n/a	Involved in the study
☒	☐ Antibodies
☒	☐ Eukaryotic cell lines
☒	☐ Palaeontology and archaeology
☒	☐ Animals and other organisms
☒	☐ Clinical data
☒	☐ Dual use research of concern
☒	☐ Plants

Methods

n/a	Involved in the study
☒	☐ ChIP-seq
☒	☐ Flow cytometry
☐	☒ MRI-based neuroimaging

Plants

Seed stocks: n/a

Novel plant genotypes: n/a

Authentication: n/a

Magnetic resonance imaging

Experimental design

Design type: Resting-state

Design specifications: Rest consisted of one session where participants were resting in the scanner with their eyes closed for 8 minutes and 40 seconds.

Behavioral performance measures: N/A

Acquisition

Imaging type(s): Functional and structural

Field strength: 3T

Sequence & imaging parameters: High-resolution structural images were obtained using a T1-weighted magnetisation-prepared rapid sequence (MPRAGE; TE= 2.99 ms; TR= 2250ms; TI = 900 ms; voxel size = 1x1x1 mm; field-of-view = 256x240x192 mm; flip angle = 9°). Resting-state fMRI data was acquired using a T2*-weighted gradient echo planar image (EPI) sequence (32 slices; TE = 30 ms, TR = 1970ms, voxel size: 3x3x4.44 mm; field-of-view: 192x192 mm, slice thickness = 3.7mm, flip angle = 78°).

Area of acquisition: Whole brain

Diffusion MRI ☐ Used ☒ Not used

Preprocessing

Preprocessing software: All preprocessing was conducted in the CONN toolbox using its default pipeline (RRID:SCR_009550; version 22.a). Functional MRI data were first realigned and unwarped using SPM12 (Andersson et al., 2001). For this, all scans were co-registered to the first volume using a least-squares approach and a 6-parameter rigid body transformation (Friston et al., 1995). These were then resampled using b-spline interpolation to correct for motion and magnetic susceptibly interactions. Temporal misalignment between slices was corrected using the SPM12 slice-timing correction procedure (Henson et al., 1999; Sladky et al., 2011), which involved sinc temporal interpolation to resample each BOLD timeseries slice to a common mid-acquisition time. Potential outlier scans (based on motion and global signal fluctuation) were identified using conservative (95th percentile) outlier parameters in ART (Artifact Detection Tools; Whitfield-Gabrieli et al., 2011). Specifically, volumes with framewise displacement above 0.5 mm, or global BOLD signal changes above 3 SDs, were flagged as outliers (Nieto-Castanon, 2022; Power et al., 2014). A reference mean BOLD image was then computed for each subject by averaging all scans, excluding outliers. Finally, functional data was smoothed using a Gaussian kernel of 5 mm full-width half-maximum (FWHM; e.g., Dombrovski et al., 2020).

Normalization: Following this, functional and structural images were separately normalised into standard MNI space and segmented into

Normalization

grey matter, white matter and CSF 'tissue' types using the SPM12 unified segmentation and normalization algorithm (Ashburner and Friston, 2005; Ashburner, 2007). Functional and structural images were then resampled to 2 mm and 1 mm isotropic voxels, respectively, following a direct normalization procedure (Calhoun et al., 2017; Nieto-Castanon, 2022) with the default IXI-549 tissue probability map template.

Normalization template

MNI152

Noise and artifact removal

Following preprocessing, the fMRI data were next denoised using the default pipeline in the CONN toolbox (Nieto-Castanon, 2020). This involved regressing out noise components using an anatomical component-based noise correction procedure (aCompCor), which included noise components from white matter (5 noise components), CSF (5 noise components), motion parameters (3 translational and 3 rotational and their first order derivatives; Friston et al.,1996), outliers volumes derived from scrubbing (38 factors) (Power et al., 2014), effect of rest and its first order derivatives (2 factors; default setting which removes residual trends/instabilities only at the beginning of the timeseries). These were followed by a bandpass frequency filtering of the BOLD timeseries (Hallquist et al., 2013) between 0.01 Hz and 0.09 Hz (e.g., Stefanov et al., 2020), which filters low frequencies (e.g., physiological noise).

Volume censoring

Volumes with framewise displacement above 0.5 mm were flagged as outliers and removed.

Statistical modeling & inference

Model type and settings

A first-level seed-based connectivity analysis was conducted using the CONN toolbox. Here, the BOLD timeseries for each hippocampal seed (left hippocampus, right hippocampus, anterior hippocampus, posterior hippocampus), as well as confound regressors (e.g., white matter and CSF) were entered as first-level covariates into general linear models (GLMs). Functional connectivity between the seeds and every other voxel in the brain was represented by the Fisher-transformed bivariate correlation coefficient (r-to-Z) from a weighted GLM. At the first level, we conducted eight analyses whereby four of them related to single seed and four of them to between-seed contrasts. Here we considered the issue of shared variance, and this was minimised by the use of separate GLMs for each seed which avoids multicollinearity between seeds in the same model. For the single seed analyses, the left, right, anterior, and posterior hippocampus design matrices contained a single column representing all subjects, allowing the investigation of seed-to-voxel connectivity for each ROI in isolation.

Effect(s) tested

To examine hemispheric and long-axis differences in hippocampal connectivity with the cerebellum, we specified between-seed contrasts (referred to as between-source contrasts in CONN). Here, the design matrices included two columns representing each seed in each contrast. Contrasts compared the connectivity of left and right hippocampus (contrast vectors: 1 -1 and -1 1). Others compared the connectivity of anterior and posterior hippocampus (contrast vector: 1 -1 and -1 1). For single seed analyses, beta images were carried to the second-level analysis for one-sample t-tests. For between-seed analyses, contrast images generated in the first-level analysis were carried over to the second-level analysis for one-sample t-test. To explore age-related variation in hippocampal-cerebellar functional correlations, we also re-specified these GLMs with age as an additional subject-level effect, firstly as a regressor-of-no-interest (contrast vector: 1 0), and then as a regressor-of-interest (contrast vector: 0 1) into a bivariate regression analysis. For these contrasts, age was mean-centred and to ensure that we captured anticorrelations, we applied a contrast vector of 0 -1 to the demeaned age data.
To additionally control for cardiovascular/metabolic health, which has been shown to influence functional connectivity (e.g., Rashid et al., 2023; Park et al., 2018; Beyer et al., 2017), we re-specified GLMs of age for a subset of the participants (due to unavailability of BMI for the full number of participants) with BMI (demeaned) and sex (binarized, 1 = female, 0 = male) (contrast vector 0 -1 0 0) as covariates-of-no-interest. We also conducted GLMs with BMI (contrast vectors: 0 0 -1 0 and 0 0 1 0) and sex (0 0 -1 0 and 0 0 1 0) as covariates-of-interest, controlling for age.

Specify type of analysis: ☐ Whole brain ☒ ROI-based ☐ Both

Anatomical location(s)

Regions of interests (ROIs) for the hippocampus were defined using probabilistic, anatomical atlases. Hippocampal seed ROIs were created by combining the hippocampal ROI from the Harvard-Oxford subcortical atlas and the subiculum ROI from the Jülich histological atlas (Amunts et al., 2005) ensuring that the hippocampal ROI was extended medially incorporating the subicular complex (see Hodgetts et al., 2017). The Harvard-Oxford atlas ROI was thresholded at 50% and the Jülich atlas ROI thresholded at 75% to ensure both ROIs were constrained to grey matter and did not extend into adjacent regions. Using this method, left and right hippocampal ROIs were defined (Figure 1a). For the long-axis analysis, the hippocampal ROIs were split into anterior and posterior zones arbitrarily at the uncal apex (Hodgetts et al., 2017; Poppenk et al., 2013), corresponding to MNI slice y = -21 (see Figure 1b for segmentation of the left hippocampus).

Statistic type for inference

(See Eklund et al. 2016)

Voxel-wise (uncorrected p<0.001) and cluster-wise at (FWEp<0.05).

Correction

The unthresholded group-level t-statistic maps (as well as between-source contrast maps) from CONN were interrogated in SPM. These were thresholded using a family-wise error correction p-FWE <0.05 based on Random Field Theory (Worsley et al., 1996). The thresholded images were then loaded into SUIT to localise (and visualise) significant clusters within cerebellar subregions.

Models & analysis

n/a | Involved in the study
- [ ] | [x] Functional and/or effective connectivity
- [x] | [ ] Graph analysis
- [x] | [ ] Multivariate modeling or predictive analysis

Functional and/or effective connectivity: Pearson correlations.
